# Analysis of* LRRK2*,* SNCA,* and* ITGA8* Gene Variants with Sporadic Parkinson's Disease Susceptibility in Chinese Han Population

**DOI:** 10.1155/2016/3474751

**Published:** 2016-09-07

**Authors:** Jie Fang, Kehui Yi, Mingwei Guo, Xingkai An, Hongli Qu, Qing Lin, Min Bi, Qilin Ma

**Affiliations:** ^1^Department of Neurology, The First Affiliated Hospital of Xiamen University, Xiamen, China; ^2^The First Clinical Medical College of Fujian Medical University, Fuzhou, China; ^3^Department of Neurology, The First Affiliated Hospital of Gannan Medical University, Ganzhou, China

## Abstract

*Background*. Parkinson's disease (PD) is an age-related neurodegenerative disease affected by multiple genetic and environmental factors. We performed a case-control study on candidate gene to scrutinize whether genetic variants in* LRRK2*,* SNCA*, and* ITGA8* genes could be associated with sporadic PD in Chinese Han population.* Methods*. Five single-nucleotide polymorphisms (SNPs) of* LRRK2* (rs1491942),* SNCA* (rs2301134, rs2301135, and rs356221), and* ITGA8* (rs7077361) were selected and genotyped among 583 unrelated PD patients and 558 healthy controls.* Results*. Rs1491942 of* LRRK2* gene had a significantly higher genotype frequency (*P* = 3.543E − 09) and allelic G/C frequencies (*P* = 2.601E − 10) in PD patients than controls. Rs2301135 of* SNCA* gene also showed an obvious difference in genotype frequency (*P* = 4.394E − 07) and allelic G/C frequencies (*P* = 9.116E − 13) between PD patients and controls. SNPs rs2301134 and rs356221 of* SNCA* gene and rs7077361 of* ITGA8* gene lacked the significant association with the susceptibility of PD in Chinese Han population.* Conclusions*. Our study firstly expresses that rs1491942 of* LRRK2* and rs2301135 of* SNCA* gene are substantially associated with sporadic Parkinson's disease in Chinese Han population.

## 1. Introduction

Parkinson's disease (PD), the second most common neurodegenerative disease after Alzheimer's disease, consists of two major pathological hallmarks: loss of dopaminergic neurons and the presence of Lewy bodies (LB). The classic manifestations of PD are characterized by resting tremor, rigidity, bradykinesia, and impairment of postural reflexes. In addition, some untypical nonmotor features, such as sleep disturbances, mood disorders, autonomic dysfunction, sensory problems, and cognitive impairment, are highly concerned recently. Even when treated with effective therapies, PD is progressive and somehow leads to disability or even mortality.

Increasing evidence supports that complex factors contribute a lot to the susceptibility of PD, which includes genetic and environmental factors [1–3]. In the past decades, a large number of Genome-Wide Association Studies (GWAS), Candidate Gene Replication Study (CGRS), and subsequent meta-analysis studies have found that a number of potential genes and single-nucleotide polymorphisms (SNPs) associated with PD, including both risk variants and protective variants [4]. In addition, the previous candidate genetic studies provided conclusive evidence showing SNPs in* LRRK2*,* SNCA*, and* ITGA8* genes significantly impact PD susceptibility and disease characteristics.

Several variations of* LRRK2* gene were identified as risk factors for PD. For example, rs34778348 (G2385R, c.7153G>A) and rs33949390 (R1628P, c.4883G>C) were seen to associate with PD in Asian population [5, 6]. Another novel SNP within* LRRK2*, rs1491942, was found to be responsible for PD in Caucasian populations [7, 8]. However, it was never reported in Chinese Han population before.

*SNCA*, as the first pathogenic gene identified in PD, encodes *α*-synuclein, the primary component of LB, the pathological hallmark of PD. From then on, several SNPs of* SNCA* were highly considered as the genetic risk factors for sporadic PD. Located in the promoter region of* SNCA*, two SNPs (rs2301134 and rs2301135) with high allele frequency were reported in some studies as PD-related SNPs in European and Taiwanese cohorts [9, 10]. One SNP (rs356221) in the 3′UTR region of* SNCA* gene showed association with susceptibility to sporadic PD in Japanese and Taiwanese cohorts [10, 11]. All these three SNPs of* SNCA* (rs2301134, rs2301135, and rs356221) have not been investigated in Han population on the Mainland of China.

While* ITGA8* (encoding integrin alpha 8, a type-I transmembrane protein) gene was firstly proved to connect with idiopathic PD in Caucasian population in Simón-Sánchez's study [4], it was not featured as a PD relevant gene until Lill's study revealed its potential association with PD [8]. Additional studies are needed to screen the potential pathogenic variants within this gene and assess the potential role of these variants in PD pathogenesis.

There are no study that explores the association of the three genes and their SNPs with Parkinson's disease in Chinese Han population. Here, we perform the first SNP replication study on previously published SNPs within* SNCA* (rs356221, rs2301134, and rs2301135),* LRRK2* (rs1491942), and* ITGA8* (rs7077361) gene in Chinese Han population to explore the ethnic differences and recognize predictive factors for the diagnosis of PD.

## 2. Methods

### 2.1. Subjects

This study recruits 1136 cases in the Neurology Department of the First Affiliated Hospital of Xiamen University, which includes 583 Chinese Han sporadic PD patients and 553 matched healthy controls. PD diagnosis coincided well with the diagnostic criteria of UK Parkinson's Disease Society Brain Bank [12]. Among all the PD patients, the mean age is 65.10 ± 8.90 and the ratio of male to female patients is 320 : 263. The group of controls consists of healthy volunteers from the Medical Center of the First Affiliated Hospital of Xiamen University, the mean age of which is 65.37 ± 9.03, and the ratio of male to female patients is 286 : 267 (Table 1). All subjects are Han population, and the two groups are matched for age, gender, ethnicity, and area of residence. Moreover, this study has gained approval of the local ethics committees, and all patients and controls signed informed consents.

### 2.2. Genetic Analysis

Venous blood specimens are collected directly from all PD patients and the healthy controls with ethylene diamine tetraacetic acid (EDTA) anticoagulant. Genomic DNA is extracted from the blood samples with the QIAamp DNA Mini Kit (Qiagen, Hilden, Germany) under the manufacturer's instructions and stored at −20°C. The five SNPs are genotyped by Multiplex Snapshot technique (Applied Biosystems by Life Technologies, Foster City, CA, USA). The primers are designed for each SNP locus using Primer 5 and listed in Table 2. Multiplex PCR reactions are performed to amplify target regions containing the selected SNPs. All products are analyzed by the ABI PRISM 3730 DNA Sequence, of which the sequence analyses are conducted by DNA Sequencing Analysis software, GeneMapper4.0. To confirm the results, 10% patients and 10% controls are randomly selected for Sanger sequencing approaches. The concordance rate for replicate approaches was 100%.

### 2.3. Statistical Analysis

The statistical analyses are processed with SPSS, version 20.0 (IBM, Armonk, NY, USA). The clinical data are expressed as the means ± standard deviation (SD) for the continuous variables and as numbers (percentage) for the quantitative variables. Student's* t*-test is used to compare the age variables between the patients and controls. The gender variables are assessed by the chi-square test. Differences in frequencies of the alleles and genotypes between cases and controls are tested for each SNP through Pearson's chi-square test and Fisher's exact test. The criterion for significance is set at *P* < 0.05 based on two sides for all of the tests. The statistical power is calculated by Power and Sample Size Calculations version 3.1.2. The Hardy-Weinberg equilibrium (HWE) is tested by adopting the public statistics web tool (http://ihg.gsf.de/cgi-bin/hw/hwa1.pl). The Haploview program [13] is used for the calculation of linkage disequilibrium (LD) among the three SNPs in* SNCA.*

## 3. Results 

Genotype and allele frequencies of each SNP of all 1136 subjects (583 patients and 553 healthy controls) are shown in Table 3. Among PD patients, 131 (22%) had an early age of onset (<50 years) and around 77% of the patients were LOPD (≧50 years). The age (*P* = 0.860) and gender (*P* = 0.284) show no statistical difference between the PD patients and the controls in our study. All the subjects were ethnic Hans.

Linkage disequilibrium between the* SNCA* SNPs rs2301134 and rs356221 is *r* ^2^ = 0.235, while for rs356221 and rs2301135 it is *r* ^2^ = 0.066 and for rs2301134 and rs2301135 it is *r* ^2^ = 0.127. This shows weakly correlation with in rs2301134, rs2301135, and rs356221.

Single marker analysis showed a number of significantly statistical associations in our study. Two SNPs from* SNCA* and* LRRK2* genes displayed *P* values < 0.05 prior to correction, both of which were estimated ORs > 1.4. The best *P* value SNPs from both* LRRK2* and* SNCA* genes were further analyzed for age stratification.

Between all PD patients and controls,* LRRK2* gene showed a significant difference in the genotype frequency of variant rs1491942 (*P* = 3.543E − 09) and allelic G/C frequencies (*P* = 2.601E − 10, OR = 1.884, and 95% CI: 1.55–2.30). In the three SNPs of* SNCA* gene, only variant rs2301135 met the statistics standard in genotype frequencies (*P* = 4.39E − 07) and the allelic G/C frequencies (*P* = 9.116E − 13, OR = 7.857, and 95% CI: 4.05–15.26). The* ITGA8* variant rs7077361 failed to show significant difference in this group (*P* > 0.05).

In the subgroup of EOPD patients and the controls (age < 50 years), the differences were still obvious in the genotype frequencies of variant rs1491942 of* LRRK2* gene (*P* = 1.200E − 02) and allelic G/C frequencies (*P* = 3.028E − 03, OR = 1.924, and 95% CI: 1.24–2.98). Three SNPs of* SNCA* gene (*P* > 0.05) and one SNP of* ITGA8* gene (*P* > 0.05) failed to show significant difference in this group. Our statistic data of the LOPD patients and controls aged ≧ 50 years also surpassed the significance thresholds. The variant rs1491942 of* LRRK2* gene showed a *P* value of 2.538E-07 in genotype frequencies. And the allelic G/C frequencies have a *P* value of 2.459E-08 while the OR is 1.874, and the 95% CI ranged from 1.50 to 2.34. The difference is obvious in the variant rs2301135 of* SNCA* gene in the genotype frequencies (*P* = 5.561E − 07) and allelic G/C frequencies (*P* = 1.45E − 12, OR = 7.846, and 95% CI: 4.03–15.29) in the subgroup. All SNPs in our study met Hardy-Weinberg equilibrium except for the rs2301135 of* SNCA* gene, shown in Table 3.

## 4. Discussion 

Since the first Genome-Wide Association Study on sporadic Parkinson's disease was performed in 2005, a new era starts to gain attention in the genetic basis of Parkinson's disease [14]. Advances in genotyping technology and meta-analysis have allowed researchers to rapidly identify common variants related to PD in different populations [4]. Though some of the previously nominated PD risk genes were firstly reported in familiar Parkinson's disease (such as* SNCA* and* LRRK2*), both of them were successfully replicated in unrelated sporadic PD patients [15]. Additional associated studies and subsequent meta-analysis contributed a lot to identifying the unnoticed variants that can also drive PD risk,* ITGA8* as an example [8].

*LRRK2* gene was firstly featured as a PD-related gene in Zimprich's study of families with autosomal-dominant, late-onset Parkinsonism in 2004 [16]. Variants in different domains of* LRRK2* have been identified in both familial and sporadic PD in different populations [17–19]. Rs1494942 in* LRRK2* was previously found associated with PD in US and European series [8, 20]. Our result, being consistent with previous studies, suggests that polymorphism rs1491942 of* LRRK2* is a risk loci of sporadic PD, and the variant carriers may share a similar pathomechanism in different populations. As it is reported,* LRRK2* variant carriers share similar clinical and pathological features, including a wild range of onset ages, typical Parkinsonism presentation, and sensitivity to L-dopa therapy [21]. In our PD patients, the rs1491942 shows significantly higher frequencies in both EOPD and LOPD subgroups compared with matched controls. These suggest that rs1491942 does not influence the onset age but contributes to the pathogenesis of EOPD and LOPD in a similar way.

*SNCA* is the first identified causal gene in familial PD [22]. Our findings are partly consistent with previous reports of the association of polymorphisms in* SNCA* with the susceptibility of PD in US, Norway, and Italian studies [23–25]. The linkage disequilibrium of three SNPs of* SNCA* gene showed that those SNPs are independent. Only one SNP near the promoter region (rs2301135) shows significant differences between PD patients and the controls in Chinese Han population. Concerning the age of onset, rs2301135 is more likely to associate with late-onset PD in our study, while another study in UK suggested that* SNCA* risk alleles for PD may associate with earlier onset of PD [26]. Given that genotype frequencies and allelic frequencies of rs2301135 of* SNCA* gene do not follow the Hardy-Weinberg equilibrium in our study, this suggests the possibility of inappropriate population stratification and selection or other confounding factors in our study. Therefore, these results should be interpreted carefully.

*ITGA8* expressing in brain mediates cell-cell interactions and regulates neurite outgrowth of sensory and motor neurons [8].* ITGA8* gene was firstly shown associated with PD in Caucasian population in Simón-Sánchez's study but failed to replicate in other studies of Greece, Irish, and Polish series [4, 19, 20].* ITGA8* variant rs7077361 showed no evidence of relation to PD in our population. In patients and controls, the observed MAFs of the SNP rs7077361 were similar to those reported in the 1000 genomes Southern Han Chinese (CHS) population. However, there is insufficient power to detect the association of rs7077361 with PD in the current sample size. The lack of association of the rs7077361 in Chinese Han population could be ascribed to the limited sample size and the rare existence of this SNP in Chinese population. Large-sample trials from multicenter are required to better understand the contribution of rs7077361 in* ITGA8* to PD susceptibility.

In conclusion, the present study provides considerable evidence to support the significant influence of genetic variants on PD risk. It does not replicate the susceptibility of rs2301134 and rs356221 in* SNCA* and rs7077361 in* ITGA8* for PD but confirms that single-nucleotide polymorphisms rs1491942 of* LRRK2* and rs2301135 of S*NCA* gene are susceptible to sporadic PD in Chinese Han population. Certain variants are responsible for the incidence of this disease, while other variants may modify the onset age, which suggests that distinct aspects of PD have a specific genetic architecture. Further studies are required to enrich genetic architecture of PD.

## Figures and Tables

**Table 1:** Demographic characteristics of Parkinson's disease (PD) cases and controls.

Characteristics	Cases = 583		Controls = 553	
Gender (*n*, %)^*∗*^				
Female	320	54.9	286	51.7
Male	263	45.1	267	48.3
Age at collection (mean, SD)^†^	65.10	8.90	65.37	9.03
Age at onset (*n*, %)				
<50	131	22.5	n.a.	n.a.
≧50	452	77.5	n.a.	n.a.

All the subjects were ethnic Hans; ^*∗*^PD compared with controls by gender: *P* > 0.05; ^†^PD compared with controls by age: *P* > 0.05; n.a.: not applicable.

**Table 2:** PCR and Snapshot probe primer sequences.

Polymorphisms	Primers	Sequence 5′ → 3′
LRRK2		
rs1491942	Forward	CAGGCTTGGGCAATTTCTAA
	Reverse	GCCTATTGTGCTTCCTGCTC
	Probe	40Ts+CAGGCTCCCCTGGGTT

SNCA		
rs2301134	Forward	ATCACGCTGGATTTGTCTCC
	Reverse	CACGGTCACAGGTTACAACG
	Probe	41Ts+GACTCTTCCTTAGTAGTCTCCC
rs356221	Forward	TGCCATAGAAACAACGAGGA
	Reverse	TTGAAGAACCCAAAATGCAA
	Probe	24Ts+AAGAGAAGCCATCCTAGT
rs2301135	Forward	ACTTAACGTGAGGCGCAAAA
	Reverse	CGTCCTCCTCCTCCTAGTCC
	Probe	54Ts+CCGGGAGAGGGGCGGG

IGTA8		
rs7077361	Forward	TGCGAAAACTATTTGGTGAAA
	Reverse	CCCACCCACCAAATCTCTAA
	Probe	31Ts+GAAATCATCTAGGGGATA

**Table 3:** Comparison of the genotype frequencies and the allele frequencies of *LRRK2*, *SNCA*, and *ITGA8* polymorphisms.

Gene SNP	Group	Genotype *n*%						*P*_HWE_	Allele Min/Maj	MAF^a^	MAF^b^	OR (95% CI)	*P*	Power^c^
		CC		GC		GG			G/C					
*LRRK2*														
rs1491942	Patients total	291	49.9	240	41.2	52	8.9	0.84	344/822	0.30	0.36	1.884 (1.546–2.297)	2.60*E* − 10^*∗*^	1.00
	Controls total	372	67.3	161	29.1	20	3.6	0.67	201/905	0.18				
	EOPD	65	49.6	54	41.2	12	9.2	0.84	78/184			1.924 (1.244–2.976)	3.03*E* − 03^*∗*^	
	Controls < 50 y	74	68.5	29	26.9	5	4.6	0.33	39/177					
	LOPD	226	50.0	186	41.2	40	8.8	0.82	266/638			1.874 (1.500–2.340)	2.46*E* − 08^*∗*^	
	Controls ≧ 50 y	298	67.0	132	29.7	15	3.4	0.87	162/728					

		GG		AG		AA			A/G					

*SNCA*														
rs2301134	Patients total	437	75.0	132	22.6	14	2.4	0.29	160/1006	0.14	0.20	1.237 (0.964–1.588)	9.43*E* – 02	0.42
	Controls total	436	78.8	108	19.5	9	1.6	0.40	126/980	0.11				
	EOPD	97	74.0	32	24.4	2	1.5	1.00	36/226			1.561 (0.875–2.786)	1.29*E* − 01	
	Controls < 50 y	88	81.5	20	18.5	0	0.0	0.59	20/196					
	LOPD	340	75.2	100	22.1	12	2.7	0.16	124/780			1.176 (0.891–1.552)	2.52*E* − 01	
	Controls ≧ 50 y	348	78.2	88	19.8	9	2.0	0.25	106/784					

		AA		TA		TT			T/A					

rs356221	Patients total	210	36.0	282	48.4	91	15.6	0.86	464/702	0.40	0.40	1.167 (1.985–1.382)	7.50*E* − 02	0.45
	Controls total	231	41.8	244	44.1	78	14.1	0.31	400/706	0.36				
	EOPD	43	32.8	67	51.1	21	16.0	0.59	109/153			1.286 (0.887–1.864)	1.84*E* − 01	
	Controls < 50 y	46	42.6	47	43.5	15	13.9	0.67	77/139					
	LOPD	167	36.9	215	47.6	70	15.5	1.00	355/549			1.135 (0.938–1.374)	1.93*E* − 01	
	Controls ≧ 50 y	185	41.6	197	44.3	63	14.2	0.36	323/567					

		CC			GG				G/C					

rs2301135	Patients total	544		93.3	39		6.7	<0.05	78/1088	0.07	0.19	7.857 (4.046–15.258)	9.12*E* − 13^*∗*^	1.00
	Controls total	548		99.1	5		0.9	<0.05	10/1096	0.01				
	EOPD	129		98.5	2		1.5	<0.05	4/258			1.016 (1.000–1.031)	1.30*E* − 01	
	Controls < 50 y	108		100.0	0		0.0	<0.05	0/216					
	LOPD	415		91.8	37		8.2	<0.05	74/830			7.846 (4.026–15.288)	1.45*E* − 12^*∗*^	
	Controls ≧ 50 y	440		98.9	5		1.1	<0.05	10/880					

		TT			CT				C/T					

*ITGA8*														
rs7077361	Patients total	580		99.5	3		0.5	1.00	3/1163	0.00	0.00	2.85 (0.296–27.443)	6.25*E* − 01	0.34
	Controls total	552		99.8	1		0.2	1.00	1/1105	0.00				
	EOPD	131		100.0	0		0.0	<0.05	0/262			0.995 (0.986–1.004)	4.52*E* − 01	
	Controls < 50 y	107		99.1	1		0.9	1.00	0/0					
	LOPD	449		99.3	3		0.7	1.00	3/449			1.003 (1.000–1.007)	2.50*E* − 01	
	Controls ≧ 50 y	445		100.0	0		0.0	<0.05	0/445					

EOPD: early onset Parkinson's disease; LOPD: late-onset Parkinson's disease; *P*_HWE_: *P* value obtained in the Hardy-Weinberg equilibrium (HWE) test; *∗*: significant *P* value obtained in the case-control analysis; Min: minor; Maj: major; MAF: minor allele frequency; a: this study; b: 1000 genomes (Southern Han Chinese); c: power was calculated by Power and Sample Size Calculations version 3.1.2.

## References

[1] Nalls M. A., Pankratz N., Lill C. M. (2014). Large-scale meta-analysis of genome-wide association data identifies six new risk loci for Parkinson's disease. *Nature Genetics*.

[2] McCormack A. L., Thiruchelvam M., Manning-Bog A. B. (2002). Environmental risk factors and Parkinson's disease: selective degeneration of nigral dopaminergic neurons caused by the herbicide paraquat. *Neurobiology of Disease*.

[3] Kalinderi K., Bostantjopoulou S., Fidani L. (2016). The genetic background of Parkinson's disease: current progress and future prospects. *Acta Neurologica Scandinavica*.

[4] Simón-Sánchez J., Schulte C., Bras J. M. (2009). Genome-wide association study reveals genetic risk underlying Parkinson's disease. *Nature Genetics*.

[5] Farrer M. J., Stone J. T., Lin C.-H. (2007). Lrrk2 G2385R is an ancestral risk factor for Parkinson's disease in Asia. *Parkinsonism & Related Disorders*.

[6] Ross O. A., Wu Y.-R., Lee M.-C. (2008). Analysis of Lrrk2 R1628P as a risk factor for Parkinson's disease. *Annals of Neurology*.

[7] Nalls M. A., Plagnol V., Hernandez D. G. (2011). Imputation of sequence variants for identification of genetic risks for Parkinson's disease: a meta-analysis of genome-wide association studies. *The Lancet*.

[8] Lill C. M., Roehr J. T., McQueen M. B. (2012). Comprehensive research synopsis and systematic meta-analyses in Parkinson's disease genetics: the PDgene database. *PLoS Genetics*.

[9] Winkler S., Hagenah J., Lincoln S. (2007). *α*-Synuclein and Parkinson disease susceptibility. *Neurology*.

[10] Wu-Chou Y.-H., Chen Y.-T., Yeh T.-H. (2013). Genetic variants of SNCA and LRRK2 genes are associated with sporadic PD susceptibility: a replication study in a Taiwanese cohort. *Parkinsonism and Related Disorders*.

[11] Mizuta I., Satake W., Nakabayashi Y. (2006). Multiple candidate gene analysis identifies *α*-*synuclein* as a susceptibility gene for sporadic Parkinson's disease. *Human Molecular Genetics*.

[12] Calne D. B., Snow B. J., Lee C. (1992). Criteria for diagnosing Parkinson's disease. *Annals of Neurology*.

[13] Barrett J. C., Fry B., Maller J., Daly M. J. (2005). Haploview: analysis and visualization of LD and haplotype maps. *Bioinformatics*.

[14] Maraganore D. M., De Andrade M., Lesnick T. C. (2005). High-resolution whole-genome association study of Parkinson disease. *The American Journal of Human Genetics*.

[15] Ross O. A., Gosal D., Stone J. T. (2007). Familial genes in sporadic disease: common variants of *α*-synuclein gene associate with Parkinson's disease. *Mechanisms of Ageing and Development*.

[16] Zimprich A., Biskup S., Leitner P. (2004). Mutations in LRRK2 cause autosomal-dominant parkinsonism with pleomorphic pathology. *Neuron*.

[17] Ozelius L. J., Senthil G., Saunders-Pullman R. (2006). LRRK2 G2019S as a cause of Parkinson's disease in Ashkenazi Jews. *The New England Journal of Medicine*.

[18] Lesage S., Dürr A., Tazir M. (2006). LRRK2 G2019S as a cause of Parkinson's disease in North African Arabs. *The New England Journal of Medicine*.

[19] Kara E., Xiromerisiou G., Spanaki C. (2014). Assessment of Parkinson's disease risk loci in Greece. *Neurobiology of Aging*.

[20] Soto-Ortolaza A. I., Heckman M. G., Labbé C. (2013). GWAS risk factors in Parkinson's disease: LRRK2 coding variation and genetic interaction with PARK16. *American Journal of Neurodegenerative Disease*.

[21] Dächsel J. C., Farrer M. J. (2010). LRRK2 and Parkinson disease. *Archives of Neurology*.

[22] Lesage S., Brice A. (2009). Parkinson's disease: from monogenic forms to genetic susceptibility factors. *Human Molecular Genetics*.

[23] Davis A. A., Andruska K. M., Benitez B. A., Racette B. A., Perlmutter J. S., Cruchaga C. (2016). Variants in GBA, SNCA, and MAPT influence Parkinson disease risk, age at onset, and progression. *Neurobiology of Aging*.

[24] Myhre R., Toft M., Kachergus J. (2008). Multiple alpha-synuclein gene polymorphisms are associated with Parkinson's disease in a Norwegian population. *Acta Neurologica Scandinavica*.

[25] Trotta L., Guella I., Soldà G. (2012). SNCA and MAPT genes: independent and joint effects in Parkinson disease in the Italian population. *Parkinsonism and Related Disorders*.

[26] Spencer C. C., Plagnol V., Strange A. (2011). Dissection of the genetics of Parkinson's disease identifies an additional association 5′ of SNCA and multiple associated haplotypes at 17q21. *Human Molecular Genetics*.

